# Understanding the Role of Patient Portals in Fostering Interprofessional Collaboration Within Mental Health Care Settings: Mixed Methods Study

**DOI:** 10.2196/44747

**Published:** 2023-07-19

**Authors:** Keri Durocher, Hwayeon Danielle Shin, Brian Lo, Sheng Chen, Clement Ma, Gillian Strudwick

**Affiliations:** 1 Campbell Family Mental Health Research Institute Centre for Addiction and Mental Health Toronto, ON Canada; 2 Arthur Labatt Family School of Nursing Western University London, ON Canada; 3 School of Health, Community Services, and Creative Design Lambton College Sarnia, ON Canada; 4 Institute of Health Policy, Management, and Evaluation University of Toronto Toronto, ON Canada

**Keywords:** mental health, patient portal, mixed methods, interprofessional collaboration, communication, self-empowerment, nursing informatics, digital health

## Abstract

**Background:**

Patient portals are web-based systems through which patients can access their personal health information and communicate with their clinicians. The integration of patient portals into mental health care settings has been evolving over the past decade, as cumulated research to date has highlighted the potential role of portals in facilitating positive health outcomes. However, it is currently unknown whether portal use can foster interprofessional collaboration between clinicians and patients or whether the portal is a tool to support an already established collaborative relationship.

**Objective:**

This mixed methods study aimed to understand how the use of a patient portal within mental health settings can impact the level of interprofessional collaboration between clinicians and patients.

**Methods:**

This study was conducted in a large mental health care organization in Ontario, Canada. A convergent mixed methods design was used, where the primary data collection methods included questionnaires and semistructured interviews with patients who had experience using a portal for their mental health care. For the quantitative strand, participants completed the Health Care Communication Questionnaire and the Self-Empowerment subscale of the Mental Health Recovery Measure at 3 time points (baseline, 3 months of use, and 6 months of use) to measure changes in scores over time. For the qualitative strand, semistructured interviews were conducted at the 3-month time point to assess the elements of interprofessional collaboration associated with the portal.

**Results:**

For the quantitative strand, 113 participants completed the questionnaire. For the Health Care Communication Questionnaire scores, the raw means of the total scores at the 3 time points were as follows: baseline, 43.01 (SD 7.28); three months, 43.19 (SD 6.65); and 6 months, 42.74 (SD 6.84). In the univariate model with time as the only independent variable, the scores did not differ significantly across the 3 time points (*P*=.70). For the Mental Health Recovery Measure scores, the raw mean total scores at the 3 time points were as follows: baseline, 10.77 (SD 3.63); three months, 11.09 (SD 3.81); and 6 months, 11.10 (SD 3.33). In the univariate model with time as the only independent variable, the scores did not differ significantly across the 3 time points (*P*=.34). For the qualitative strand, 10 participants were interviewed and identified various elements of how interprofessional collaboration can be supplemented through the use of a patient portal, including improved team functioning, communication, and conflict resolution.

**Conclusions:**

Although the quantitative data produced nonsignificant findings in interprofessional collaboration scores over time, the patients’ narrative accounts described how the portal can support various interprofessional collaboration concepts, such as communication, leadership, and conflict resolution. This provides useful information for clinicians to support the interprofessional relationship when using a portal within a mental health setting.

**International Registered Report Identifier (IRRID):**

RR2-10.1136/bmjopen-2018-025508

## Introduction

### Patient Portals

A patient portal is a web-based system through which patients can access their personal health information and collaborate with their health care providers [1]. Around 2006, in North America, portals started to become widely adopted into health care settings through several private initiatives [2]. Since then, the integration of patient portals into various health disciplines and care areas has shown positive health care delivery outcomes, including improved quality of care and enhanced health status [3]. Specifically, positive health outcomes have been linked to care in the management of chronic diseases [4], cancer [5], and diabetes [6]. With such vast applications in multiple health contexts, research on portal applications within specific care areas, such as mental health care, is warranted.

### Mental Health Care and Use of Portals

Over the last decade, research on patient portal integration into mental health care settings has evolved to build a body of knowledge on how to best support individuals within this broad health context. This research included various domains associated with the portal to understand the specific nuances of the evolving digital technology. Specifically, Etingen et al [7] performed a retrospective analysis through the Veterans Health Administration to determine whether individuals with specific diagnoses were more or less likely to access the portal. The researchers discovered that having anxiety disorders, posttraumatic stress disorder, and depression were associated with a greater likelihood of portal use [7]. Alternatively, Kipping et al [8] evaluated the benefits of implementing a portal for patients with mental illnesses [8]. Some noted benefits included a significant increase in appointment attendance and subjectively reported increases in autonomy [8]. Strudwick et al [9] studied various predictors of mental health professionals’ perceptions of using portals, such as their beliefs on whether patients should have portal access and whether they experience discomfort with this practice. Researchers discovered that perceptions of patient portal integration varied among different disciplines, such as psychiatrists reporting more negative perceptions of patient portals [9].

### Interprofessional Collaboration

One notable gap in this evolving body of research is understanding how a patient portal can support interprofessional collaboration between mental health care providers, patients, and family members or caregivers. Although there is significant variation in the way interprofessional collaboration has been previously defined in the literature [10], we explored the concept of when ≥2 parties form a team, including clinicians, patients, and families or caregivers, and work concurrently to meet a desired outcome through shared power and partnerships [11]. Some of the key components of interprofessional collaboration include communication, role clarification, conflict resolution, leadership, team functioning, and patient-centered care, as defined by the Canadian Interprofessional Health Collaborative (CIHC) National Interprofessional Competency Framework [12]. This framework places the patient as central to the interprofessional collaborative partnership, which helps enhance joint decision-making [12]. Within mental health settings, previous research has linked treatment adherence with effective collaborative patient-clinician relationships [13]. However, it is currently unknown whether the use of a patient portal can enhance interprofessional collaboration between clinicians, patients, and families or caregivers or whether it is simply a tool to support an already established collaborative relationship.

### Purpose and Research Objectives

The aim of this mixed methods study was to evaluate and understand the impact of patient portal use on the level of interprofessional collaboration from the perspectives of patients. The quantitative and qualitative objectives were as follows:

1. Quantitative strand: To determine whether the use of a patient portal has an impact on the level of interprofessional collaboration between patients and health care providers over time.
2. Qualitative strand: To understand how the use of a patient portal can influence patients’ perceptions of interprofessional collaboration with their health care providers.

## Methods

### Setting

This study was conducted within a large center that delivers mental health care in Toronto, Ontario, called The Centre for Addiction and Mental Health (CAMH). CAMH is the largest mental health teaching hospital in Canada, which delivers care to >34,000 patients per year across various inpatient and outpatient programs [14]. Globally, it is one of the world’s leading research institutions for mental health care.

### Patient Portal

Several versions of patient portals exist across hospitals with slightly different functionalities. There are many similarities between different portals, but MyCare has been customized to the needs of CAMH and is only being used at CAMH. Through MyCare (Cerner patient portal), patients can access personal health information in collaboration with their health care team members. Patients can access parts of their electronic health record such as demographics, laboratory results, and clinician-written notes (eg, admission or discharge and assessments). Other features of the portal include a secure clinician-patient messaging system and the ability to view upcoming appointments. These portals were integrated to select the outpatient service settings.

### Design

This research is a part of a larger study that was completed over a 2-year period, and a protocol was previously published [15]. The primary data collection methods included questionnaires and semistructured interviews with patients and family members who had experience using a portal for their mental health care implemented at the organization.

This study included a secondary analysis of previously collected interview transcripts for the larger study, with a focus on interprofessional collaboration between patients and clinicians when using the portal. For a more in-depth explanation of the methods, please refer to the original protocol [15]. Publication of the larger study is currently in progress. The data were integrated at the design level using a fixed, convergent mixed methods study design [16,17]. Qualitative and quantitative data were independently gathered during a similar time frame and then compared to gain a further understanding of the topic of interest and participants’ experiences [16,17].

### Recruitment

The participants were recruited using various techniques. One strategy was the distribution of recruitment flyers within the pamphlet that described how to use the portal. Interested participants were then provided with a link after registering for the portal, where recruitment information for the study was presented at the end of the email. Alternatively, when potential participants signed into the portal, the same recruitment information was included on the home page. If individuals had questions about the study, a research team member was on site within the outpatient settings during peak hours. Potential participants were also able to speak to the research team member in a private setting after they were enrolled to use the portal.

### Sampling

#### Quantitative Strand

The minimum sample size for the quantitative strand was estimated to be 100 participants based on power calculation, as indicated in the study protocol [15]. All participants were assumed to be able to read English, as all components of the portal were available in English. Inclusion criteria were as follows: (1) being aged >16 years, (2) had enrolled to use the patient portal, and (3) self-reported having access to the portal for a time frame of <2 weeks. All participants were from outpatient clinical settings and provided written informed consent via REDCap (Research Electronic Data Capture; Vanderbilt University).

#### Qualitative Strand

At the end of the 3-month period for quantitative data collection, a convenient sample of participants was interviewed to discuss their experiences while using the portal. The inclusion criteria for the interviews included being a patient who had accessed and used the portal for at least 3 months. Participants also had to complete the quantitative questionnaires at baseline (before portal use) and after 3 months of use to be eligible for interviewing.

### Data Collection

#### Quantitative Strand

All enrolled participants completed 2 questionnaires that encompassed crucial elements of interprofessional collaboration: the Health Care Communication Questionnaire (HCCQ) [18] and the Self-Empowerment subscale of the Mental Health Recovery Measure (MHRM) [19]. The questionnaires were administered via REDCap, a secure web application for collecting survey data. These questionnaires were administered at 3 time points: T0 (baseline), T1 (3 months of portal use), and T2 (6 months of portal use). Demographic information was also collected at the baseline data collection time point.

The HCCQ is a validated, 13-item scale that includes multiple elements of patients’ outpatient experiences, including problem-solving, respect, the lack of hostility, and nonverbal immediacy [18]. Each item refers to the concept of clinician communication, such as *keeping calm*, *solving patient problems*, and *eye contact*. Each item is scored on a 5-point Likert scale, with 0 meaning *not at all* to 5 meaning *very much* [18]. The maximum total score on the HCCQ is 65, with higher scores indicating more positive experiences of communication between patients and clinicians.

The MHRM is a 30-item self-report instrument [20], with all items being scored on a 5-point Likert scale (with a 0-4 range) for each associated item [19]. Overall scores for the MHRM can range from 0 to 120, with higher scores indicating higher levels of recovery-related experiences [21]. Self-empowerment is 1 of the 8 domains within this scale (items 5, 6, 7, and 8), and these 4 items (maximum score of 20) were analyzed in this study as a component of interprofessional collaboration.

#### Qualitative Strand

A semistructured interview guide was developed based on the objectives of the larger study. A total of 2 questions in the guide referred to interprofessional collaboration, and research assistants performed semistructured interviews using a secure videoconferencing platform (WebEx). The interviews were approximately 30 to 60 minutes in length and were completed between March 2021 and May 2022. With the consent of each participant, the interviews were audio recorded and transcribed verbatim. Any personal identifiers were removed from the transcripts before the data analysis was conducted.

### Data Analysis

#### Quantitative Strand

All quantitative data analysis procedures were performed using SAS Enterprise Guide (version 7.15; SAS Institute). Participant characteristics at baseline (T0) were summarized using descriptive statistics. Continuous measures were summarized using means and SD, whereas categorical and ordinal measures were summarized using frequencies and proportions. Linear mixed effects models with random intercepts were used to model the trajectory of each outcome across the 3 study time points. Pairwise contrasts were generated between T0 and T1, between T0 and T2, and between T1 and T2. The main analysis was not adjusted. We considered 2-sided *P* values <.05 as statistically significant.

#### Qualitative Strand

The CIHC National Interprofessional Competency Framework [12] is an established framework implemented for our secondary analysis of qualitative data. The interview transcripts were analyzed using a deductive approach [22], in which relevant domains of the framework were used. According to the CIHC, interprofessional collaboration involves active interprofessional relationships among team members, such as learners, health care professionals, and patients [12]. This CIHC framework has been implemented to study other phenomena, including interprofessional collaboration related to collaborative practice for providers [23] and advanced practice nursing [24]; however, it has not yet been applied to collaboration using a patient portal.

In total, 2 research team members (KD and HDS) were responsible for performing directed content thematic analysis of the interview data [22]. Both team members were registered nurses and PhD students with multiple years of experience in performing digital and mental health research. All transcription data were entered into NVivo Pro 11 (Lumivero) to facilitate coding and analysis procedures. As a pilot exercise, both team members coded 2 transcripts and reviewed any discrepancies before coding the rest of the data. After coding the remaining transcripts, collaborative thematic analysis was performed and mapped among 5 of the 6 themes within the framework.

#### Integration

To enhance our understanding of the quantitative and qualitative data, separate findings were reviewed simultaneously by the research team to understand how components of interprofessional collaboration relate to portal use. This helped the research team understand the contextual elements of how using the patient portal may relate to the elements of interprofessional collaboration among clinicians, patients, and family members or care partners.

### Ethics Approval and Informed Consent

Ethics approval for the study was obtained from the Research Ethics Board at CAMH (REB 044/2018) and the University of Toronto (REB #40342). Written information about the study was provided to all potential participants, and an informed consent form was signed by all participants prior to being enrolled in the study.

## Results

### Quantitative Strand

#### Demographic Characteristics

A total of 113 participants were recruited for quantitative analysis. Of the 113 participants, 70 (62%) were aged between 26 and 64 years and 77 (68.1%) identified as female (Table 1). The most common diagnosis was a mood disorder, with 38.1% (43/113) of the participants reporting this. Regarding portal access, 99.1% (112/113) of the participants reported that they had daily access to the internet. Finally, on a scale of 0 to 100, participants reported their level of family or caregiver support, with a mean of 57.5 (SD 31.3).

**Table 1:** Demographic and clinical characteristics of participants (N=113).

Characteristic			Participant, n (%)
**Sex**			
	Female	77 (68.1)	
	Male	33 (29.2)	
	Prefer not to say	3 (2.7)	
**Age range (years)**			
	<25	39 (34.5)	
	26-64	70 (61)	
	≥65	4 (3.5)	
**Marital status**			
	Never married	62 (54.9)	
	Married, domestic partnership, common law	35 (31)	
	Widowed	3 (2.7)	
	Divorced or separated	11 (9.7)	
	Prefer not to answer	2 (1.8)	
**Ethnicity**			
	Black or African American	8 (7.1)	
	East Asian	10 (8.9)	
	Hispanic or Latinx	3 (2.7)	
	Indigenous	2 (1.8)	
	South Asian	6 (5.3)	
	White or European	76 (67.3)	
	Mixed heritage	4 (3.5)	
	Other	4 (3.5)	
**Diagnosis**			
	Anxiety	21 (18.6)	
	Mood disorder	43 (38.1)	
	Other	35 (31)	
	Prefer not to answer	9 (8)	
	Schizophrenia	5 (4.4)	
**Internet access**			
	Daily	112 (99.1)	
	Weekly	1 (0.9)	

#### Scales

For both the HCCQ and MHRM-Self-Empowerment scales, from the original 113 participants who had T0 scores, 84 scores were recorded at T1 and 78 scores were recorded at T2. This is because for participants who had 1 more missing item, the total scores were not calculated for the descriptive analysis.

##### HCCQ Score

**Figure 1:**
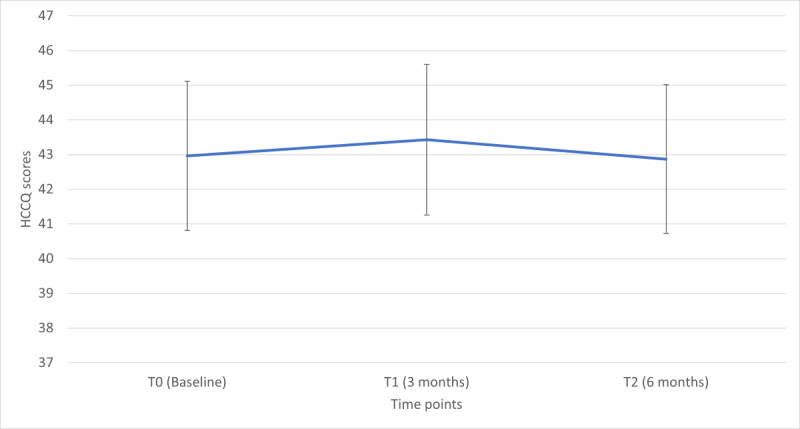
Estimated marginal means of Health Care Communication Questionnaire (HCCQ) scores over time. Error bars denote 95% CIs.

The mean total scores at the 3 time points were as follows: T0, 43.01 (SD 7.28); T1, 43.19 (SD 6.65); and T2, 42.74 (SD 6.84). In the univariate model with time as the only independent variable, the scores did not differ significantly across the 3 time points (*P*=.70). The estimated marginal means (least square means) were 42.96 (95% CI 41.60-44.33) for T0, 43.43 (95% CI 41.94-44.93) for T1, and 42.87 (95% CI 41.35-44.40) for T2 (Figure 1). Pairwise contrasts did not reveal substantial differences between T1 versus T0, T2 versus T0, and T1 versus T2. The HCCQ scores remained stable across the 3 time points.

##### Self-Empowerment Scale

**Figure 2:**
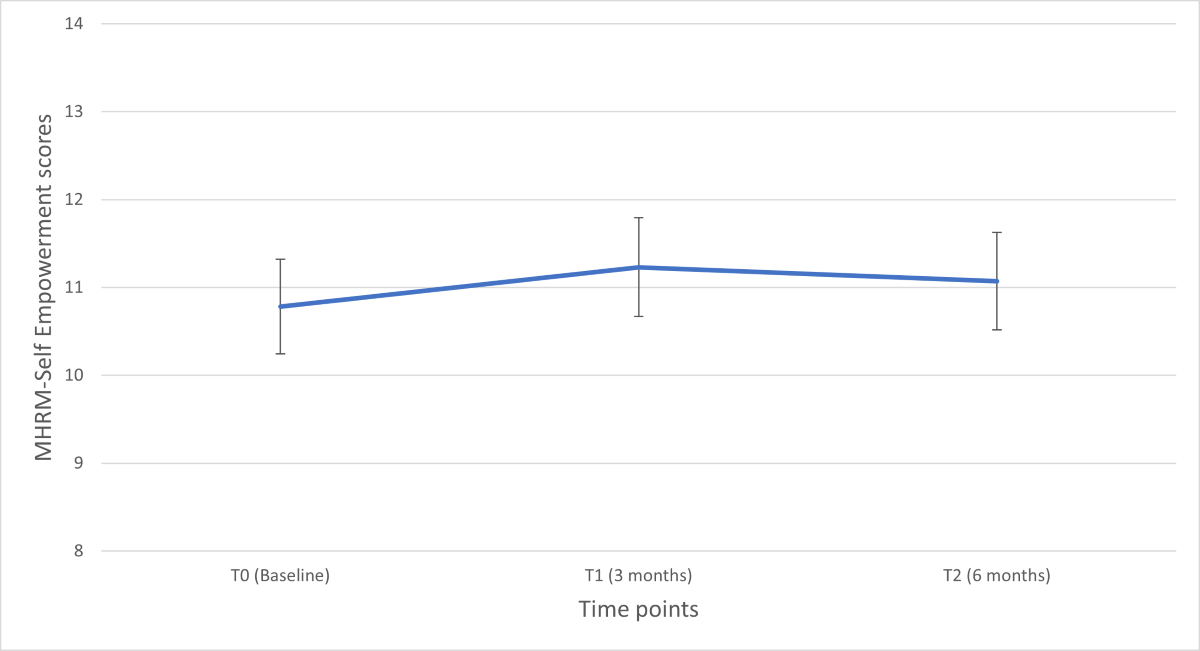
Estimated marginal means of Mental Health Recovery Measure (MHRM) Self-Empowerment subscale scores over time. Error bars denote 95% CI.

The mean total scores at the 3 time points were as follows: T0, 10.77 (SD 3.63); T1, 11.09 (SD 3.81); and T2:11.10 (SD 3.33). In the univariate model with time as the only independent variable, the scores did not differ significantly across the 3 time points (*P*=.34). The estimated marginal means (least square means) were 10.78 (95% CI 10.10-11.44) for T0, 11.23 (95% CI 10.50-11.96) for T1, and 11.07 (95% CI 10.32-11.82) for T2 (Figure 2). Pairwise comparisons of least squares means did not identify significant differences between T1 versus T0, T2 versus T0, and T1 versus T2.

### Qualitative Strand

#### Overview

In total, 11 participants completed the interviews, and 1 interview was dropped during our analysis because there were no findings related to interprofessional collaboration. The following domains of the CIHC National Interprofessional Competency Framework were identified that applied to our analysis: *Patient, Client, Family, Community-Centered Care*; *Team Functioning*; *Collaborative Leadership*; *Interprofessional Communication*; and *Interprofessional Conflict Resolution*. Exemplar participant quotes of each theme are displayed narratively in the proceeding sections, with explanations of the integration of interprofessional collaboration with the patient portal in digital mental health care. For a full list of participant data mapped to the CIHC National Interprofessional Competency Framework, please see Multimedia Appendix 1.

#### Patient, Client, Family, Community-Centered Care

Integrating portals into mental health care settings can facilitate patient-centered care by enhancing the visibility of patients’ pressing health needs. In addition, patients can review their clinical notes and verbally correct any misunderstandings or request clarification at the appointments with their clinicians. This process helps support the participation of patients and family members within the interprofessional circle of care and represents the core members of the care delivery pathway [12]. Two participants commented on how this process helped them meet their care needs and provided a sense of support and control:

I think for myself, I’m definitely the kind of person where I like being able to see the facts in front of me. I really like being able to have something written down, something concrete in front of my face, that helps me come to terms with things better, and be able to take the information and then work with it going forward.

Participant 9

So, every so often when I was on the portal there would be these surveys like, how do you feel and how do you feel about your care? Those were great. I really liked getting them when I was in recovery. I felt like I was in control of my care a lot more than without the portal.

Participant 2

#### Team Functioning

Functioning of the interprofessional team requires that all members have shared team dynamics that facilitate collaborative processes, including health care providers, patients, and their family members or care partners [12]. Through active participation, patients may feel a greater sense of control over their care outcomes. As the portal provides a channel for communication, collaborative goals can be understood by all team members, and patients may be more prepared for appointments:

I will go in more prepared with questions about...when I get a result when I’m there it’s kind of right away and you’re just trying to absorb it. So, I can check it at home, I can do my own little research, but then if I still have questions I can talk to the doctor and see what to do.

Participant 6

It felt like I was kind of in control if that makes sense... It was nice to have that come so quickly because I’m so used to talking with a doctor and it takes like six weeks to hear back from my doctor. It kind of got rid of the anxiety of having to wait. There really was no wait and it was making me feel in control of everything.

Participant 7

In addition, effective communication among the interprofessional care team can strengthen the working relationship among its members. When clinicians validate patients’ health needs and maintain ongoing communication through the use of the portal, this highlights its potential contribution to interprofessional collaboration. One participant commented on how the portal made them feel acknowledged even after their in-person appointment was finished:

I think, through the portal is kind of a way to acknowledge the fact that they are still paying it attention. They are still caring about your various health issues, whatever they may be. And it’s not like, once you leave the room, they forget about you. Not that that’s the case if you don’t have a portal, but it helps to solidify that, oh no, I am being acknowledged. My health is not being ignored, it’s right here, I’m seeing that they see it.

Participant 9

#### Collaborative Leadership

To foster excellence in care, clinicians must include patients and family members or caregivers in a collaborative practice model [12]. In doing so, patients play a key role in their care responsibilities and can inquire about areas that must be clarified by clinicians. In addition, integration of the portal into practice can minimize the need for extra appointments for care areas that can be addressed through active portal use:

When I get a result when I’m there it’s kind of right away and you’re just trying to absorb it. So, I can check it at home, I can do my own little research, but then if I still have questions I can talk to the doctor and see what to do.

Participant 6

I think it would reduce their need to do a lot of unnecessary paperwork. Let’s say they could write a prescription for some kind of drug and simply post it on the portal for the patient to print out and take to the drug store instead of, again, physically going to see the doctor, making the appointment, waiting in line, and doctors are always late. Basically, wasting a lot of everybody’s time just to get a piece of paper to take to a store when it can be accessed online. And the same thing, the instancy of information is a really great thing because it creates a good venue of communication between the patient and the doctor, not simply limited to the physical appointment.

Participant 4

Collaborative leadership also shifts the responsibility of care to a joint approach between patients, families or caregivers, and clinicians when using a portal. Therefore, clinicians must be cognizant of reducing the use of medical jargon to promote a digital environment for shared leadership [12] and ensure that patients are aware of certain medical terms and information:

I like when you talk to me as if I’m a colleague that you’re talking to and leave it to me to say to you, I don’t know what that term means, I don’t know what that definition is, and then you can backtrack and say, okay, so let me inform you. I prefer you not worrying about talking over me, as opposed to insulting me by talking down to me. That’s me.

Participant 5

#### Interprofessional Communication

The process of interprofessional communication should include a collaborative and responsive approach between the clinician and patient, which can be supplemented using a portal. When patients can thoroughly understand care decisions, it can enhance the trusting relationship with their clinician. A total of 2 participants commented on how using the portal provided this sense of trust and encouragement for having care discussions:

It gave me the chance to talk to them about some of the diagnoses. If they said, how do you feel about this diagnosis of bipolar rather than this other diagnosis of schizo affective, or whatever? It was good to know where that was coming from, and it was also good to know the reasoning behind it without having to waste time during a meeting with the psychiatrist or the doctor.

Participant 2

I think a lot of people don’t trust their clinician, especially today because there’s a lot of misinformation out there and, I don’t know, people don’t always trust healthcare professionals. If you give someone access to the same information as a healthcare professional has access to then it, theoretically, would...It theoretically should increase the trust level there because I can... If I don’t think... I could look up that lab value.

Participant 1

One participant also remarked how portal implementation can improve efficiencies in communication and reduce the need for having duplicate conversations:

That’s just a good record to have of what has been covered so that we don’t need to waste the appointment time, which is usually an hour or so, fairly short, on covering things that had already been covered. It’s good for that, I would say, and basically keeping track of the progress. So, seeing the whole transition from appointment to appointment and where that leads.

Participant 4

Despite the positive aspects of how portals can enhance communication, 1 participant remarked that despite the integration of a patient portal, there may still be uncertainty regarding whether the clinician is fully forthcoming in what is placed within the portal for patient viewing. This demonstrates the importance of building a trusting, foundational relationship in addition to the implementation of supportive technology into care relationships:

So, it’s like I’m having the information relayed to me, like there’s a middleman, kind of. So, I think that there isn’t as much of a trust, necessarily. Or there’s always a bit of questioning of, well, am I getting the full story here? Am I getting the full scope of information that I need, or am I getting what they believe is all I need? So, being able to read it myself, I know that I’m being given the information because I’m seeing it in front of me. I know that what they said they do believe because they also included it for me to access.

Participant 9

#### Interprofessional Conflict Resolution

When interprofessional relationships are developed between clinicians and patients, conflict can be an inevitable component of the ongoing caring relationship. As noted in the previous quote, uncertainty regarding what is included in clinical notes can be a potential source of conflict. However, working collaboratively to build consensus on issues and actively working to solve disagreements are strategies for conflict resolution that can be supplemented using a portal [12]. Some areas to consider are the portal design components and information entered by the clinician within the notes. In total, 2 participants commented on how these design elements are important, which speaks to missed opportunities for conflict resolution:

The comment that I had about the notes is it would have been nice for me to be able to flag certain things. I had been at an inpatient facility and one of the nurses there had given an account of events about how something had occurred. I would have really appreciated the opportunity to flag that and give my interpretation, because in the portal there was only one... it was great to see what was written, but there was only one side to it.

Participant 2

Certainly, seeing doctors’ notes, what they said, may have been. Because I feel like I never really properly understood. If people paraphrase what I say, I find that they often change what I perceive is the meaning of my statement. So, if I could see someone writing notes and them not being accurate to the message I was trying to convey.

Participant 5

One participant also remarked how the implementation of the portal could reduce some sources of conflict, such as questioning the usefulness of certain assessments or interventions. If patients can understand the rationale for these activities, this source of conflict can be reduced:

I was filling out these mood charts and then he would just file them away and I questioned if he was reading them, I questioned if I was wasting my time. I feel like maybe if I was submitting them on the portal, at least I’d feel like someone is looking at them in the meantime, like, I’m submitting them before I get there.

Participant 1

### Integration

Despite a lack of change on the scales related to communication and self-empowerment over time, participants revealed many different perceptions related to interprofessional collaboration through the semistructured interviews. This demonstrates how various elements of how interprofessional collaboration relate to portal use may be best described through the subjective, narrative experiences of patients. For example, a few participants commented on how interprofessional communication practices can improve through various components of the portal, such as through a preemptive chart review. Therefore, a notable benefit of this secondary analysis is that after merging these data, we now have a deeper understanding of some aspects of interprofessional collaboration that can be enhanced by portal use and other aspects that require further exploration.

## Discussion

### Principal Findings

The aim of this mixed methods study was to evaluate and understand the impact of portal use on patients’ experiences of interprofessional collaboration within a mental health context. Previous literature describes the role of technology in collaboration from the providers’ perspectives [25,26]. At the time of writing, this study is one of the first to assess the impact of portal use on collaboration from the patients’ perspectives, who are the central members of the care team. The quantitative results showed no significant findings, whereas the qualitative strand sheds light on the impact of portal use on multiple components of interprofessional collaboration beyond clinicians’ communication skills and patients’ sense of empowerment. For example, portals encouraged patients’ participation in their own care, promoting collaborative leadership and a sense of control. Furthermore, portals helped reshape traditional team dynamics, ensuring that patients are central members of the team, in contrast to research on interprofessional teams that primarily focuses on working interactions between different providers [27,28]. Therefore, the portal’s ability to encourage participation from patients is noteworthy because ensuring the full participation of patients as interprofessional collaborators can minimize professional paternalism [29,30]. Most notably, portal use does not seem to detract from promoting interprofessional collaboration.

Despite the potential for portal use in facilitating interprofessional collaboration in mental health care settings, there were a few areas of concern that must be acknowledged. For example, trusting relationships must be established between patients and health care providers. Otherwise, patients perceived that their notes are not fully disclosed to them, which could be a barrier to establishing true coleadership of patients and providers in the care team. In addition, interpreting clinical notes can be a challenge, which consistently have been reported in the current literature in mental health care settings [31] and beyond [32]. These gaps in the current practice of interprofessional collaboration when using a portal provide foundational criteria for building future directions in mental health settings.

### Future Directions

Considering health equity factors is imperative when implementing a portal for mental health care to avoid heightening the digital divide for this patient population as well as to foster collaboration [33]. The development of approaches to bridge this divide for patients receiving mental health care should focus on strategies that promote these equitable health outcomes [34]. In total, 2 potential interventions to reduce the digital divide include further promoting family or caregiver collaboration and encouraging open review of clinical notes.

As we defined family or caregiver support as a crucial component of the interprofessional team, a future area of exploration includes how perceptions of this support relate to use of the portal and interprofessional collaboration. Within the demographic questionnaire, the average level of family support, as rated by the participants, was 57.5 on a 0 to 100 scale. With regard to mental health care, the impact of family support has been explored in recent literature related to mental health outcomes for lesbian, gay, bisexual, transgender, queer, and similar minority youth [35] and disaster recovery for children [36]. However, this has not been extensively explored in the literature related to digital mental health interventions, such as portal use, or from the perspective of including the patient or family members as a part of the team. Furthermore, Reed et al [37] explored some factors of engagement between family members and portal use, such as reviewing laboratory results and filling prescriptions. A more in-depth analysis of how interprofessional collaboration factors align with the engagement of family members or caregivers in portal use may provide mental health clinicians with insight for enhancing interprofessional collaboration.

Being able to view different types of notes through the use of a portal was a commonly identified area by the participants to help enhance the levels of interprofessional collaboration. This process can be facilitated through the OpenNotes movement in mental health care, where patients and families or caregivers can collaboratively review their health care information with their clinicians to gain a further understanding of their care trajectory [9]. One way that the use of OpenNotes can improve interprofessional collaboration is through patient empowerment and engagement [38,39], as power can be shifted and redistributed among all interprofessional team members. This may also serve to enhance the level of trust between interprofessional team members, which was a concept mentioned various times by the participants in the interviews.

### Limitations

One notable limitation of the qualitative strand of this study is that the semistructured interview guide was not produced specifically to examine interprofessional collaboration. As this secondary analysis is part of a larger study, some interview questions were tailored specifically to interprofessional collaboration, whereas other interview questions examined other factors related to the portal, such as compassion and recovery. Although responses to other questions also yielded relevant findings on this topic, future work in this space may choose to focus on additional areas of interprofessional collaboration, such as role clarification [40]. Despite being a part of the CIHC National Interprofessional Competency Framework, specific elements of role clarification between clinicians, patients, and family members or caregivers were not explored. Role clarification questions may focus on understanding individual responsibilities within an interprofessional team and being able to understand the roles of all members [12].

A limitation of the quantitative strand was the lack of validated scales that measure interprofessional collaboration or important components of this concept. Despite being aligned with the CIHC National Interprofessional Competency Framework, self-empowerment and communication may not encompass the robust elements of what defines interprofessional collaboration of clinicians, patients, and families or caregivers. Other scales have been developed that explore elements of interprofessional collaboration but only through various clinicians (eg, physicians and nurses), rather than including patients and families or caregivers as a part of the team [41]. Finally, this study was conducted in 1 mental health hospital in Canada, and most participants were White. Therefore, our findings need to be interpreted with caution, as they have limited generalizability.

### Conclusions

The integration of patient portals into mental health care has been developing over the last decade to support positive health outcomes. This secondary analysis helped us explore whether interprofessional collaboration can be supplemented through the use of a portal, specifically between clinicians, patients, and family members or caregivers. Despite nonsignificant findings from the quantitative data, narrative accounts of patients who have used a portal for their mental health care described various aspects of how it contributed to different domains of interprofessional collaboration. This provides useful information for mental health clinicians when continuing to adopt patient portals in their practice. Future work that explores these concepts related to components of health equity, such as the role of enhanced family support and collaborative note sharing, can help extend our understanding of improving portal use in mental health care in the future.

## Appendix

Multimedia Appendix 1 Thematic analysis: the Canadian Interprofessional Health Collaborative National Interprofessional Competency Framework.

## Abbreviations

CAMH: The Centre for Addiction and Mental Health
CIHC: Canadian Interprofessional Health Collaborative
HCCQ: Health Care Communication Questionnaire
MHRM: Mental Health Recovery Measure
REDCap: Research Electronic Data Capture
