# Supplementary material for: Understanding the Role of Patient Portals in Fostering Interprofessional Collaboration Within Mental Health Care Settings: Mixed Methods Study
Source: JMIR Hum Factors. 2023 Jul 19;10:e44747. doi: 10.2196/44747 (PMC10398557; doi:10.2196/44747)
Supplement: Multimedia Appendix 1 [file humanfactors_v10i1e44747_app1.docx]

**Multimedia Appendix 1: Thematic Analysis- CIHC’s National Interprofessional Competency Framework**

| **Domain** | **Definition** | **Descriptors and Related Data** |
| --- | --- | --- |
| Collaborative Leadership | Learners/practitioners understand and can apply leadership principles that support a collaborative practice model. This domain supports shared decision-making as well as leadership, but it also implies continued individual accountability for one’s own actions, responsibilities and roles as explicitly defined within one’s professional/disciplinary scope of practice. | *Establishment of a climate for collaborative practice among all participants.*  “When I get a result when I’m there it’s kind of right away and you’re just trying to absorb it. So, I can check it at home, I can do my own little research, but then if I still have questions I can talk to the doctor and see what to do.” - T6  *Facilitation of effective decision making.*  “I like when you talk to me as if I’m a colleague that you’re talking to and leave it to me to say to you, I don’t know what that term means, I don’t know what that definition is, and then you can backtrack and say, okay, so let me inform you. I prefer you not worrying about talking over me, as opposed to insulting me by talking down to me. That’s me. Other people aren’t the same.”- T5  *Work with others to enable effective patient and client outcomes.*  “I think it would reduce their need to do a lot of unnecessary paperwork. Let’s say they could write a prescription for some kind of drug and simply post it on the portal for the patient to print out and take to the drug store instead of, again, physically going to see the doctor, making the appointment, waiting in line, and doctors are always late. Basically, wasting a lot of everybody’s time just to get a piece of paper to take to a store when it can be accessed online. And the same thing, the instancy of information is a really great thing because it creates a good venue of communication between the patient and the doctor, not simply limited to the physical appointment.”- T4  “Improved efficiency can improve the client care interaction because speedy information is always, the faster the better. And I think the client will often kind of have maybe a bias unconsciously even though they might know that it’s not the responsibility of the professional, the medical professional to uphold the website. It’s more of an IT thing. It can improve the relationship by kind of allowing the interaction to happen in a quicker manner.”- T3 |
| Interprofessional Communication | Learners/practitioners from different professions communicate with each other in a collaborative, responsive and responsible manner. | *Communicate to ensure common understanding of care decisions.*  “It gave me the chance to talk to them about some of the diagnoses. If they said, how do you feel about this diagnosis of bipolar rather than this other diagnosis of schizoaffective, or whatever? It was good to know where that was coming from, and it was also good to know the reasoning behind it without having to waste time during a meeting with the psychiatrist or the doctor." -T2  *Develop trusting relationships with patients, clients, families, and other members.*  “I think a lot of people don’t trust their clinician, especially today because there's a lot of misinformation out there and, I don't know, people don’t always trust healthcare professionals. If you give someone access to the same information as a healthcare professional has access to then it, theoretically, would … It theoretically should increase the trust level there because I can … If I don’t think … I could look up that lab value.”- T1  “So, it’s like I’m having the information relayed to me, like there’s a middleman, kind of. So, I think that there isn’t as much of a trust, necessarily. Or there’s always a bit of questioning of, well, am I getting the full story here? Am I getting the full scope of information that I need, or am I getting what they believe is all I need? So, being able to read it myself, I know that I’m being given the information because I’m seeing it in front of me. I know that what they said they do believe because they also included it for me to access.”-T9  *Effectively use information and communication technology to improve interprofessional patient-centred care.*  “That’s just a good record to have of what has been covered so that we don’t need to waste the appointment time, which is usually an hour or so, fairly short, on covering things that had already been covered. It’s good for that, I would say, and basically keeping track of the progress. So, seeing the whole transition from appointment to appointment and where that leads”. - T4  “Much easier than having to wait several weeks to hear back from a doctor, where you could have it uploaded right away, and I could see it within one to two days.” -T7 |
| Interprofessional Conflict Resolution | Learners/practitioners actively engage self and others, including the client/patient/family, in positively and constructively addressing disagreements as they arise. | *Develop a level of consensus among those with differing views.*  “The comment that I had about the notes is it would have been nice for me to be able to flag certain things. I had been at an inpatient facility and one of the nurses there had given an account of events about how something had occurred. I would have really appreciated the opportunity to flag that and give my interpretation, because in the portal there was only one … it was great to see what was written, but there was only one side to it.” T2  *Effectively working to address and resolve disagreements.*  “The vagueness with the diagnosis and stuff, instead of saying such and such a person has such and such condition, they say, presents as, or presents certain, let me quote, “endorses several symptoms consistent with”, you know? Maybe that’s how each psychiatrist speaks or writes documents, I’m not sure. I haven’t had prior experience with them. So, if I were to change anything maybe I would try to be more concrete or request the doctor to be more concrete and more direct.” T4  “Certainly, seeing doctors’ notes, what they said, may have been. Because I feel like I never really properly understood. If people paraphrase what I say, I find that they often change what I perceive is the meaning of my statement. So, if I could see someone writing notes and them not being accurate to the message I was trying to convey.” T5  *Identifying common situations that are likely to lead to disagreements or conflicts.*  ” It kind of made me feel like they didn’t, I don't know, it just made … Almost like they didn’t care as much just because they're not using all the tools at their disposal, do you know what I mean? Because I was filling out these mood charts and then he would just file them away and I questioned if he was reading them, I questioned if I was wasting my time. I feel like maybe if I was submitting them on the portal, at least I'd feel like someone is looking at them in the meantime, like, I'm submitting them before I get there.” -T1 |
| Patient, Client Family, Community-Centred Care | Learners/practitioners seek out, integrate and value, as a partner, the input, and the engagement of the patient/client/family/community in designing and implementing care/services. | *Ensure that appropriate education and support is provided to patients, family members, and others involved in care.*  “It was very descriptive. Because for this specific medication it was very detailed of how it needed to be taken, the dosage. There was a lot of information for it. And both my doctor and I actually talked about how it was so important to get that information to know exactly what to do.”- T9  *Listen respectively to the expressed needs of all parties in shaping and delivering care.*  “I don’t think it changed my opinion of the clinicians. I’m going to say that when you have a mental health issue and you’re going through recovery, it’s very normal to be paranoid about things and it’s very normal to feel like people just don’t get it. So, I remember times reading the notes and being like malcontent about it. But today, I would see those same clinicians and I would be very happy to see them. If they helped me and they understood where I was coming from, and they took the time to listen and all the stuff that you would expect from good humans.” - T2  *Share information with patients in a respectful manner and in such a way that it is understandable, encourages discussion, and enhances participation in decision-making.*  “I saw with my friend who many, many years ago had cancer and then the cancer centre allowed her to go in and get her own results. And for her it was like an empowering thing because she was part of it. That’s how she explained it, just being part of it is empowering and I think that it’s important for everyone to have that. To be able to speak up. To be able to know what’s going on with them and ask questions.” -T6  *Support the participation of patients, families, and community representatives as integral partners with healthcare personnel.*  “And it was really helpful because I could read it to my parents and they could give me feedback, they’re like, yeah, that was the way that it was. It was a useful tool for coming to terms with the schizophrenia of having two different people, one that you see of yourself and one that other people see of you. I would say, overall, it was absolutely a positive thing. To be able to have access to that information made me feel …” T2  “So, every so often when I was on the portal there would be these surveys like, how do you feel and how do you feel about your care? Those were great. I really liked getting them when I was in recovery. I felt like I was in control of my care a lot more than without the portal.” T2  “I think for myself, I’m definitely the kind of person where I like being able to see the facts in front of me. I really like being able to have something written down, something concrete in front of my face, that helps me come to terms with things better, and be able to take the information and then work with it going forward.” T9 |
| Team Functioning | Learners/practitioners understand the principles of team work dynamics and group/team processes to enable effective interprofessional collaboration. | *Effectively facilitate discussions and interactions among team members.*  “I will go in more prepared with questions about … when I get a result when I’m there it’s kind of right away and you’re just trying to absorb it. So, I can check it at home, I can do my own little research, but then if I still have questions I can talk to the doctor and see what to do.”-T6  *Establish and maintain effective and health working relationships with learners, patients, and families.*  “It felt like I was kind of in control if that makes sense. And again, it was nice just not having to dwell on something and think about it like, oh, is he trying to write some crazy think about me and say I’m completely mental. It was nice to have that come so quickly because I’m so used to talking with a doctor and it takes like six weeks to hear back from my doctor. It kind of got rid of the anxiety of having to wait. There really was no wait and it was making me feel in control of everything.” T7  “I think, through the portal is kind of a way to acknowledge the fact that they are still paying it attention. They are still caring about your various health issues, whatever they may be. And it’s not like, once you leave the room, they forget about you. Not that that’s the case if you don’t have a portal, but it helps to solidify that, oh no, I am being acknowledged. My health is not being ignored, it’s right here, I’m seeing that they see it.” -T9 |
